# Supplementary material for: Osteoclasts protect bone blood vessels against senescence through the angiogenin/plexin-B2 axis
Source: Nat Commun. 2021 Mar 23;12:1832. doi: 10.1038/s41467-021-22131-1 (PMC7987975; doi:10.1038/s41467-021-22131-1)
Supplement: Supplementary file 1 — Supplementary Information [file 41467_2021_22131_MOESM1_ESM.pdf]

Supplementary Information

Osteoclasts Protect Bone Blood Vessels Against Senescence through the Angiogenin/Plexin-B2 Axis

Xiaonan Liu, Yu Chai, Guanqiao Liu, Weiping Su, Qiaoyue Guo, Xiao Lv, Peisong Gao, Bin Yu, Gerardo Ferbeyre, Xu Cao, and Mei Wan

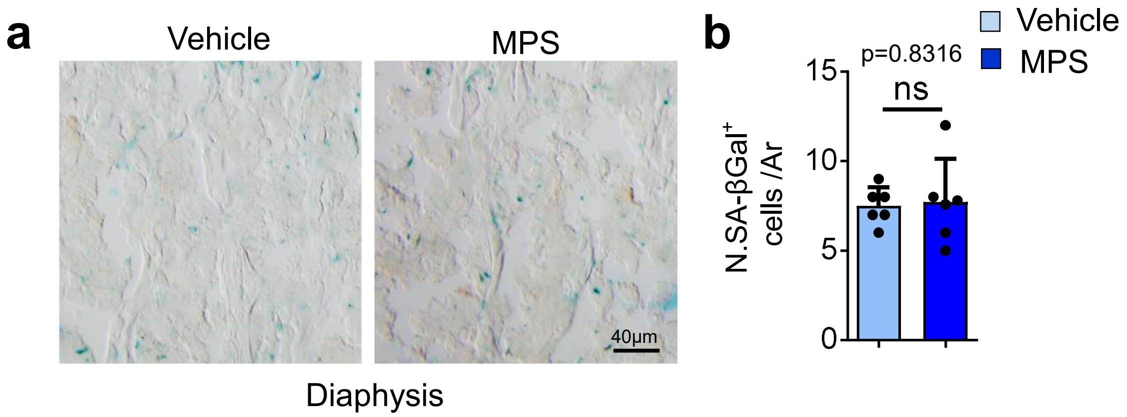

Supplementary Figure 1 Senescent Cells Were Not Detected in Diaphysis of Long Bone in Young Mice after GC Treatment. Three-week-old male *BALB/c* mice were treated with methylprednisolone (MPS, 10 mg/m^2/day) or vehicle by daily intraperitoneal injection for 3 weeks. Representative images of senescence-associated β-galactosidase (SA-βGal) staining (blue) (**a**) and quantification (**b**) of SA-βGal$^+$ cells (N. SA-βGal$^+$ cells/ Ar) in diaphysis of femoral bone. n=5-6. Data are represented as mean ± s.e.m. Ar, tissue area. ns, not significant as determined by two-tailed Student's *t*-tests.

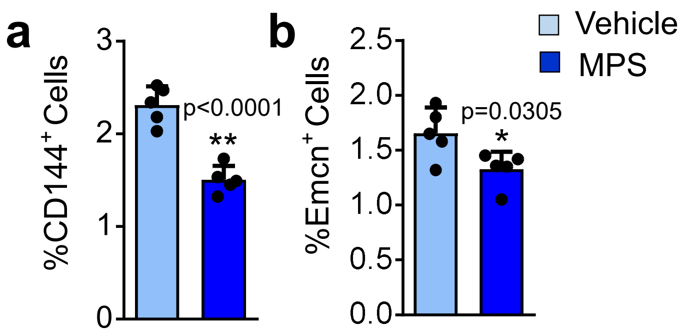

Supplementary Figure 2 Percentage of Vascular Endothelial Cells Was Decreased in Metaphysis of Long Bone in Young Mice after GC Treatment. (**a-b**) Three-week-old *BALB/c* mice were treated with methylprednisolone (MPS, 10 $mg/m^2/day$) or vehicle by daily intraperitoneal injection for 3 weeks. Percentage of $CD144^+$ or $Emcn^+$ cells was calculated by flow cytometry analysis in (**a**) and (**b**), respectively. n=5 mice. Data are represented as mean ± s.e.m. Ar, tissue area. *p< 0.05, **p< 0.01 as determined by two-tailed Student's *t*-tests.

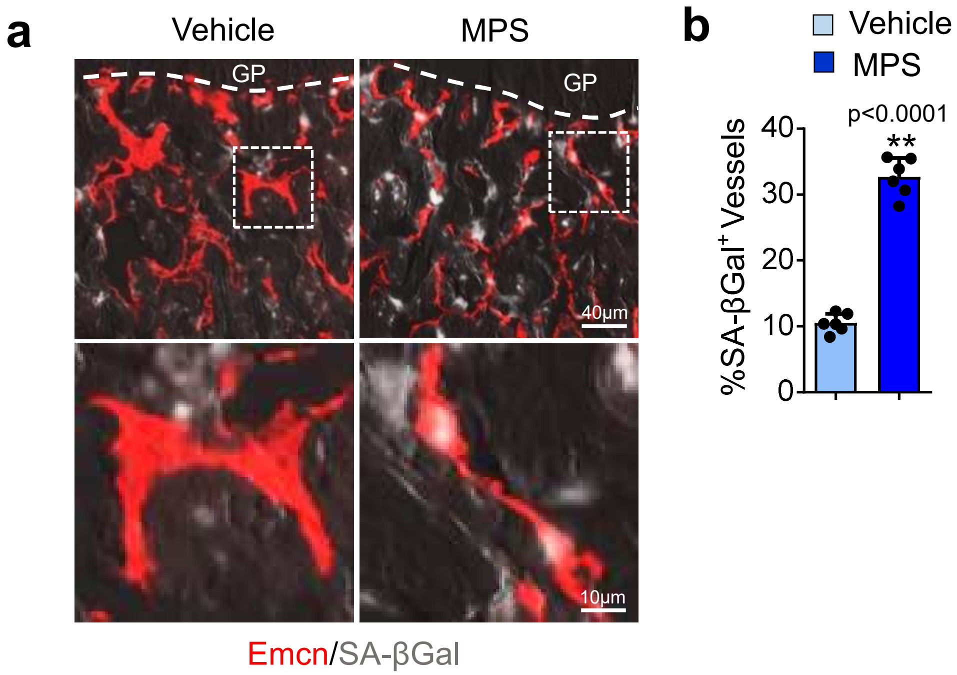

Supplementary Figure 3 Vascular Endothelial Cells Have Increased SA-βGal Activity after GC Treatment. (**a-b**) Three-week-old *BALB/c* mice were treated with MPS at 10 mg/m^2/day or vehicle by daily intraperitoneal injection for 3 weeks. Representative images of SA-βGal staining (white) and immunostaining of endomucin (Emcn, red) in primary spongiosa of femoral bone in (**a**). Images in upper panels are lower power with boxes outlining the area of higher power in bottom panels. Percentage of SA-βGal-expressing vessels in primary spongiosa was quantified in (**b**). n=6 mice. Data are represented as mean ± s.e.m. GP, growth plate. **p< 0.01 as determined by two-tailed Student's *t*-tests.

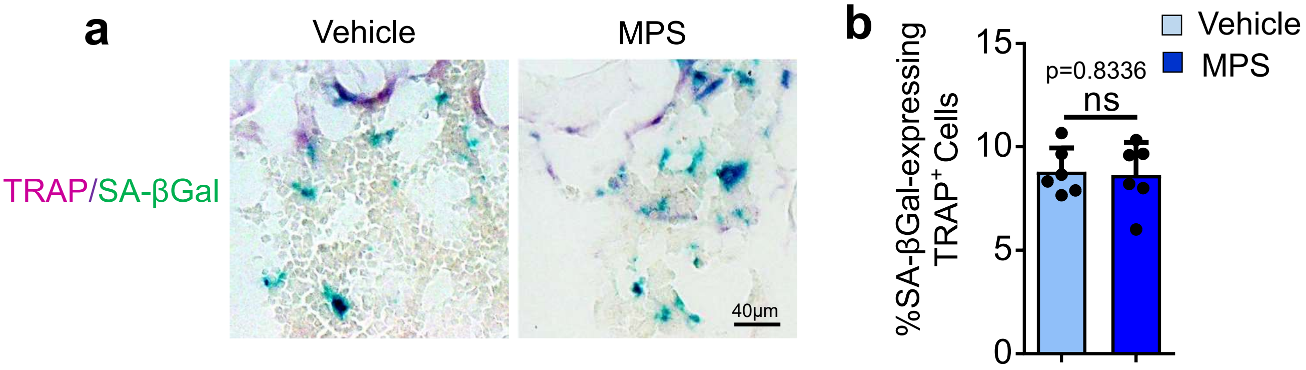

Supplementary Figure 4 Osteoclast Lineage Cells in Femoral Metaphysis Are Not the Major Senescent Cell Type in Young Mice after GC Treatment. (**a-b**) Three-week-old *BALB/c* mice were treated with MPS at 10 mg/m^2/day or vehicle by daily intraperitoneal injection for 3 weeks. TRAP- (pink) and SA-βGal- (blue) co-staining of femur metaphysis sections was performed in (**a**). Quantitative analysis of the percentage of SA-βGal-expressing TRAP$^+$ cells is shown in (**b**). n=5 mice. Data are represented as mean ± s.e.m. ns, not significant as determined by two-tailed Student's *t*-tests.

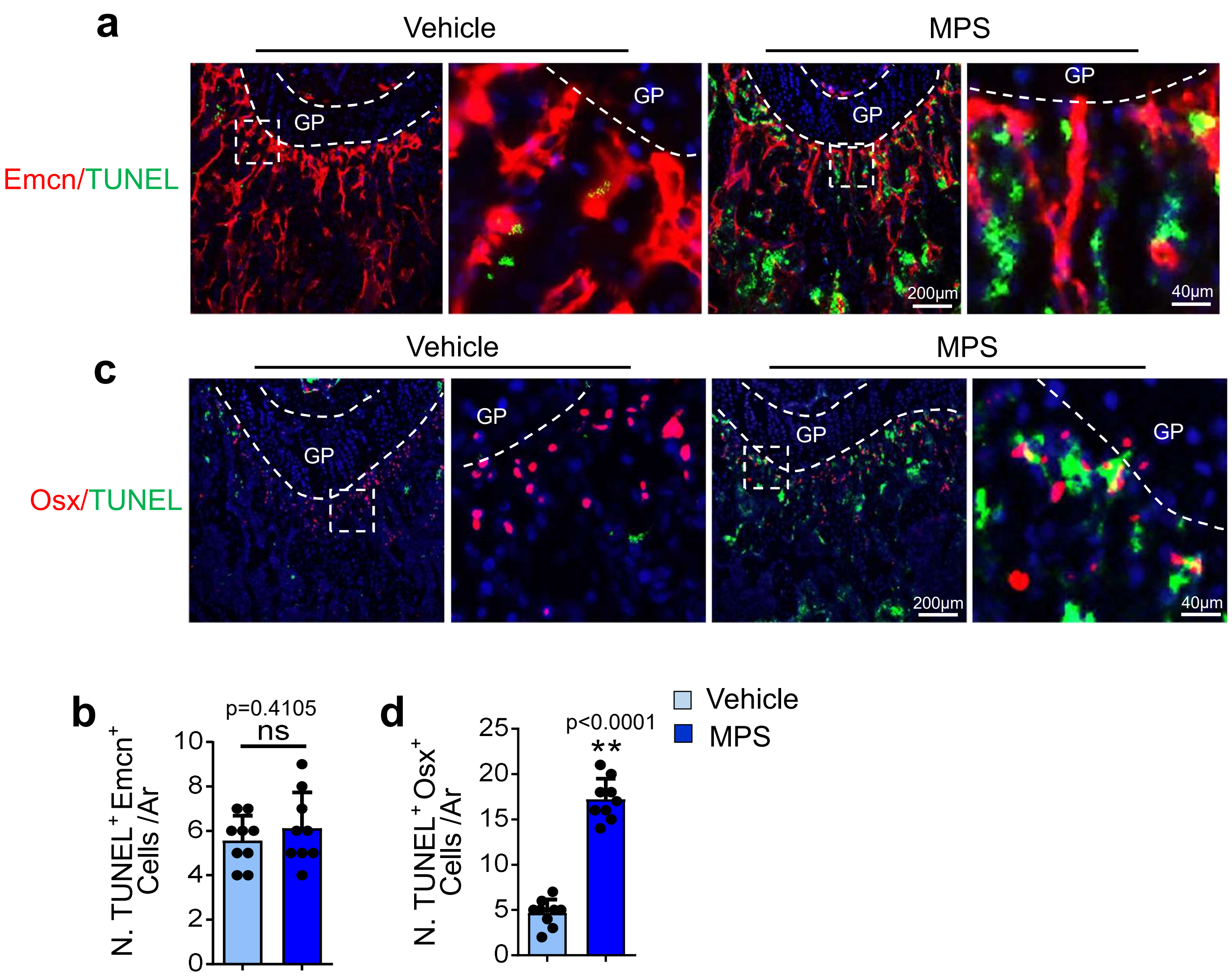

Supplementary Figure 5 Osteoprogenitors but Not Vascular Endothelial Cells Undergo Apoptosis in Femoral Metaphysis of Young Mice after GC Treatment. (**a-d**) Three-week-old *BALB/c* mice were treated with MPS at 10 mg/m^2/day or vehicle by daily intraperitoneal injection for 3 weeks. Femoral bone tissue sections were subjected to TUNEL assay and immunostaining of Emcn (**a**) or Osx (**c**). Apoptotic cells were in green and Emcn$^+$ or Osx$^+$ cells were in red. DAPI stains nuclei blue. Percentage of TUNEL$^+$Emcn$^+$ vascular endothelial cells and TUNEL$^+$Osx$^+$ osteoprogenitors were shown in (**b**) and (**d**), respectively. n=5 mice. Data are represented as mean ± s.e.m. GP, growth plate. Ar, tissue area. **p< 0.01, ns, not significant as determined by two-tailed Student's *t*-tests.

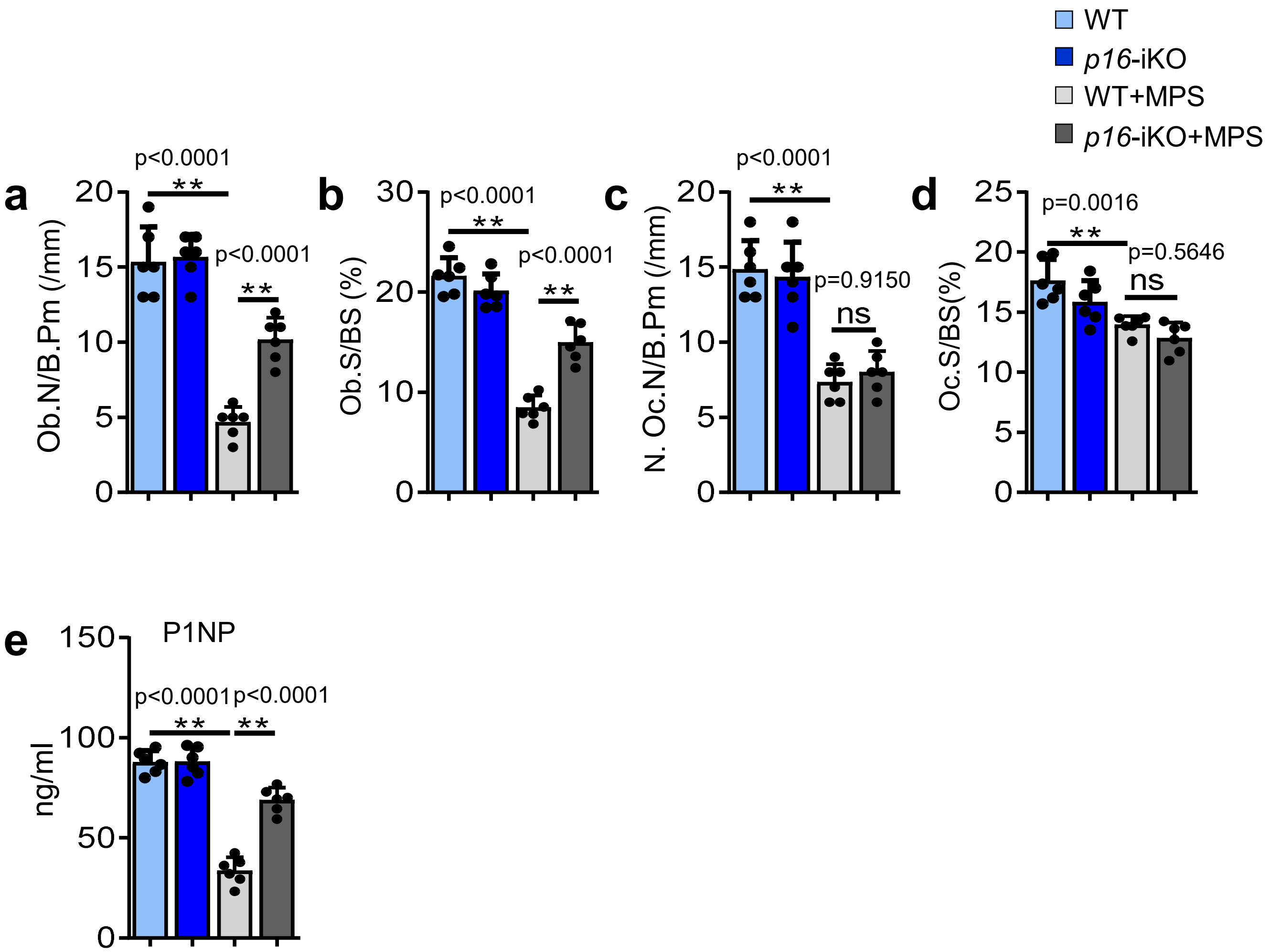

Supplementary Figure 6 Histomorphometry Analysis of Osteoblastic Bone Formation in *p16*-iKO Mice. (**a-e**) Three-week-old *Cdh5-Cre*^{ERT2};*p16*$^{flox/flox}$ (*p16*-iKO) mice and *p16*$^{flox/flox}$ (WT) mice were treated as described in Figure 3 and killed at 6 weeks of age. Bone histomorphometric analysis of femoral metaphysis was performed. Number of osteoblasts per bone perimeter (Ob.N/B. Pm) and osteoblast surface per bone surface (Ob.S/BS) were measured in (**a**) and (**b**), respectively. Number of osteoclasts per bone perimeter (Oc.N/B. Pm) and osteoclast surface per bone surface (Oc.S/BS) were measured in (**c**) and (**d**), respectively. P1NP concentration was measured in blood samples (**e**). n=5-7 mice. Data are represented as mean ±s.e.m. **p<0.01, ns, not significant as determined by one-way ANOVA with post hoc Tukey test.

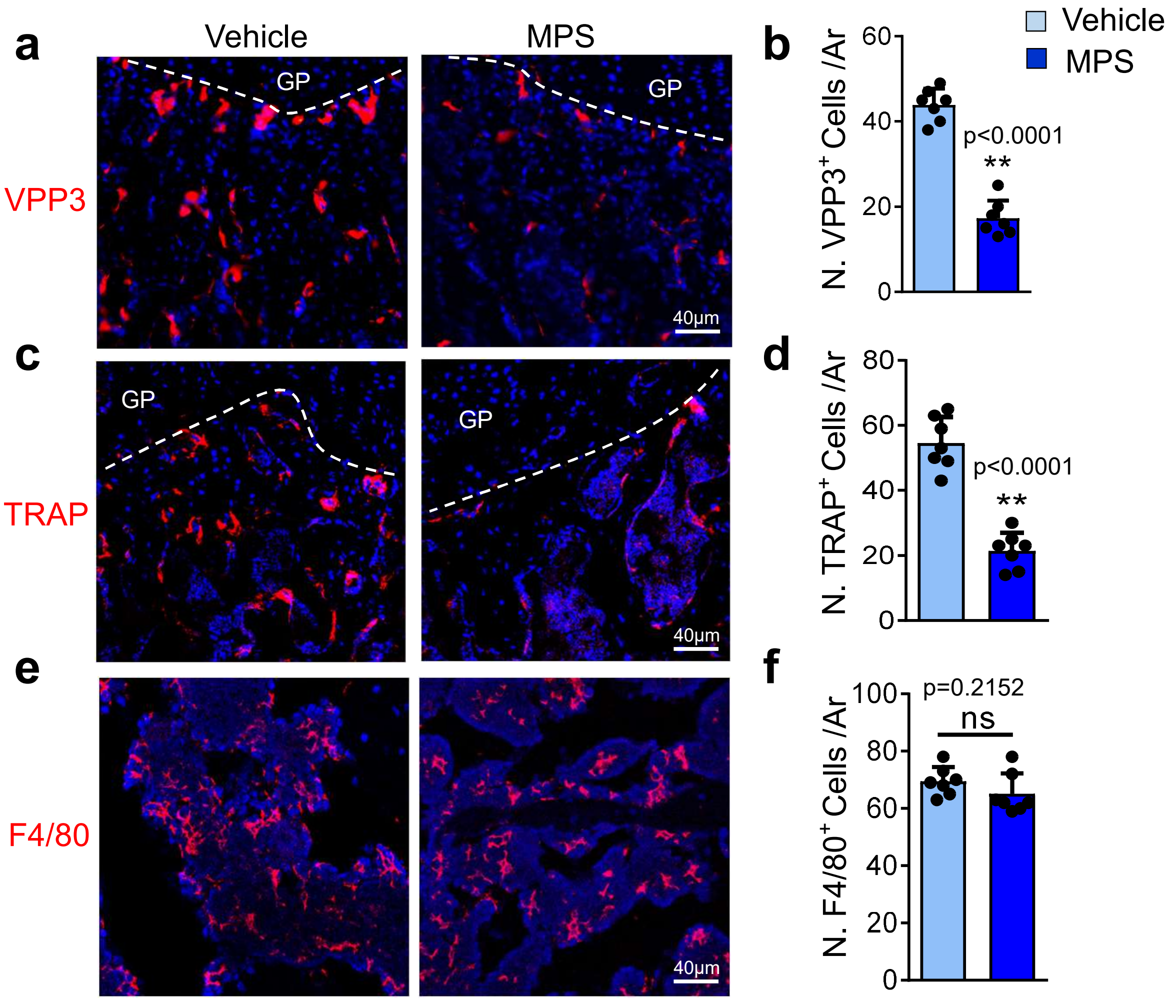

Supplementary Figure 7 GC Treatment Decreases the Number of Osteoclast-Lineage Cells in Femoral Metaphysis in Young Mice. (**a-f**) Three-week-old *BALB/c* mice were treated with MPS at 10 mg/m^2/day or vehicle by daily intraperitoneal injection for 2 weeks. Immunofluorescence staining of femoral bone sections was performed using antibodies against TRAP (**a**), VPP3 (**c**), and F4/80 (**e**), respectively. DAPI stains nuclei blue. Quantified numbers of TRAP$^+$ cells, VPP3$^+$ cells, and F4/80$^+$ cells in metaphysis are shown in (**b**), (**d**) and (**f**), respectively. GP, growth plate. Ar, tissue area. n=5-6 mice. Data are represented as mean ±s.e.m. **p< 0.01, ns, not significant as determined by two-tailed Student's *t*-tests.

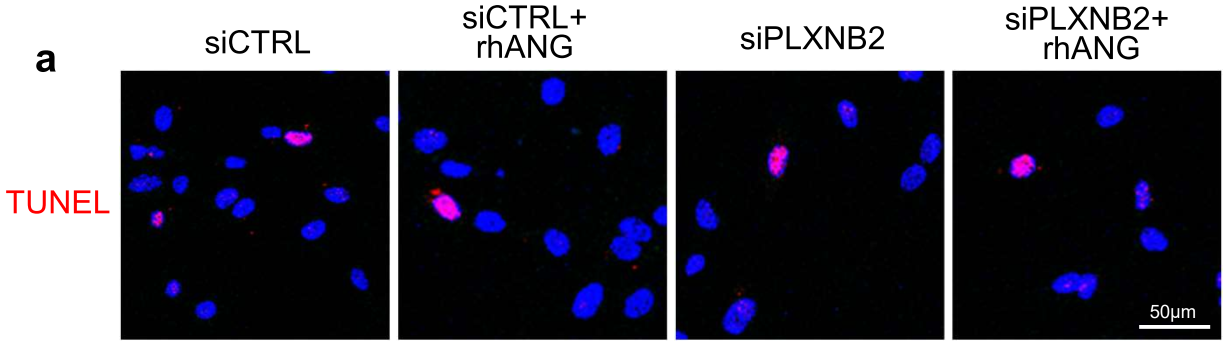

Supplementary Figure 8 Apoptosis of Endothelial Cells Were Not Induced by PLXNB2 Knockdown. (**a**) HUVECs were transfected with scrambled control siRNA (siCTRL) or PLXNB2 specific siRNA (siPLXNB2). Thirty-six hours later, the cells were treated with 200 ng/ml rhANG or vehicle for another 12 hours. TUNEL assay was performed, and the apoptotic cells are shown in red.

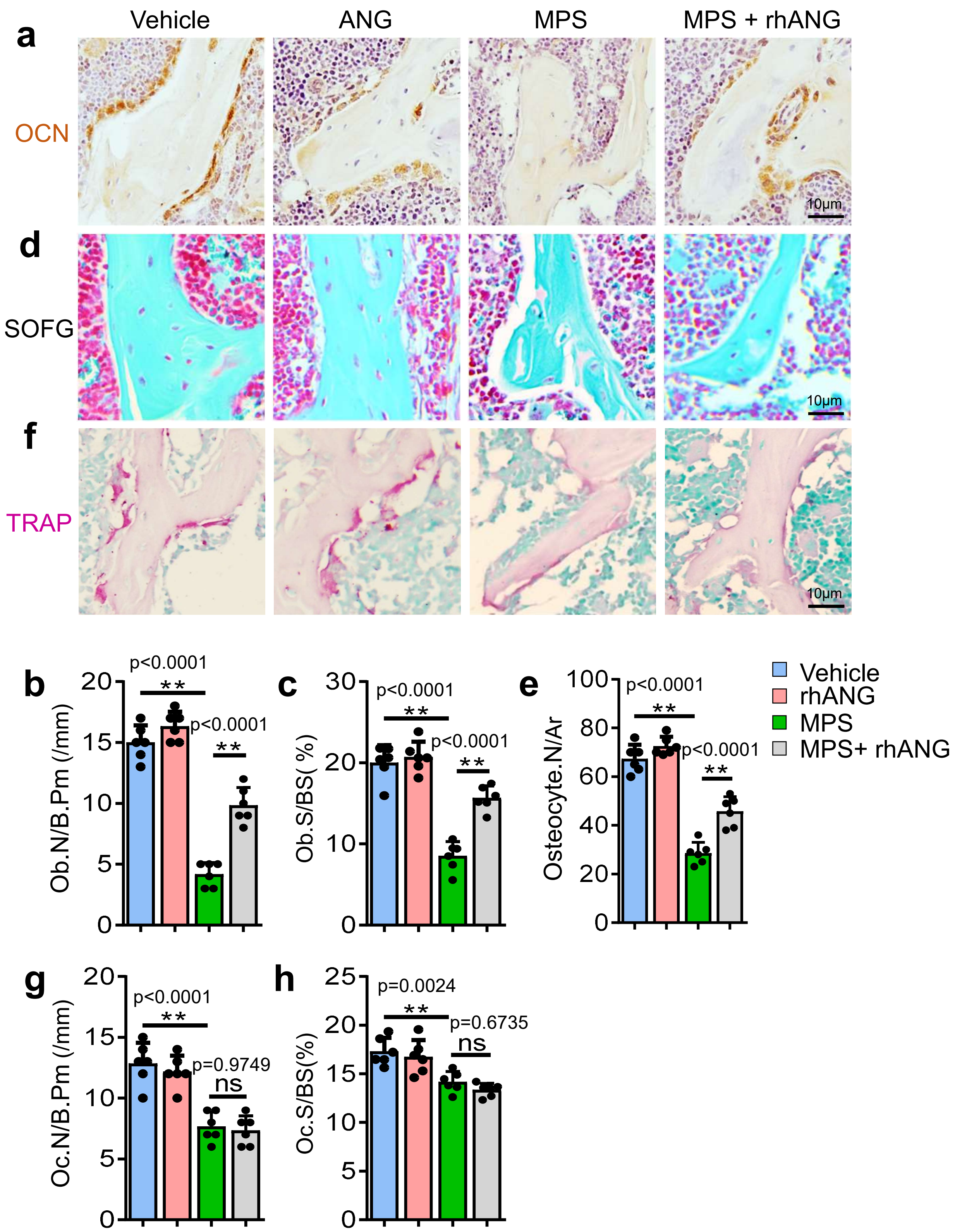

a
Vehicle
ANG
MPS
MPS + rhANG
OCN
d
SOFG
f
TRAP
10μm
b
Ob.N/B.Pm (/mm)
p<0.0001
**
p<0.0001
**
c
Ob.S/BS(%)
p<0.0001
**
p<0.0001
**
e
Osteocyte.N/Ar
p<0.0001
**
p<0.0001
**
Vehicle
rhANG
MPS
MPS+ rhANG
g
Oc.N/B.Pm (/mm)
p<0.0001
**
p=0.9749
ns
h
Oc.S/BS(%)
p=0.0024
**
p=0.6735
ns

Supplementary Figure 9 rhANG Attenuates GC-impaired Osteogenesis in Femoral Metaphysis in Young Mice. (**a-h**) Three-week-old *BALB/C* mice were treated with vehicle, MPS alone at 10 mg/m^2/day or MPS plus rhANG (1 μg/day) by daily intraperitoneal injection for 4 weeks. Representative images of OCN^+ osteoblasts on trabecular bone surface in (**a**). Number of osteoblasts per bone perimeter (Ob.N/B. Pm) and osteoblast surface per bone surface (Ob.S/BS) were measured in (**b**) and (**c**), respectively. Representative images of Safarin O-fast green (SOFG) staining in primary spongiosa of femoral bone in (**d**). Number of osteocytes(Osteocyte.N/Ar) were quantified in (**e**). Representative images of $TRAP^+$ osteoclasts on trabecular bone surface in (**f**). Number of osteoclasts per bone perimeter (Oc.N/B. Pm) and osteoclast surface per bone surface (Oc.S/BS) were measured in (**g**) and (**h**), respectively. n=6-10 mice. Data are represented as mean ±s.e.m. **$p<0.01$, ns, not significant as determined by one-way ANOVA with post hoc Tukey test.

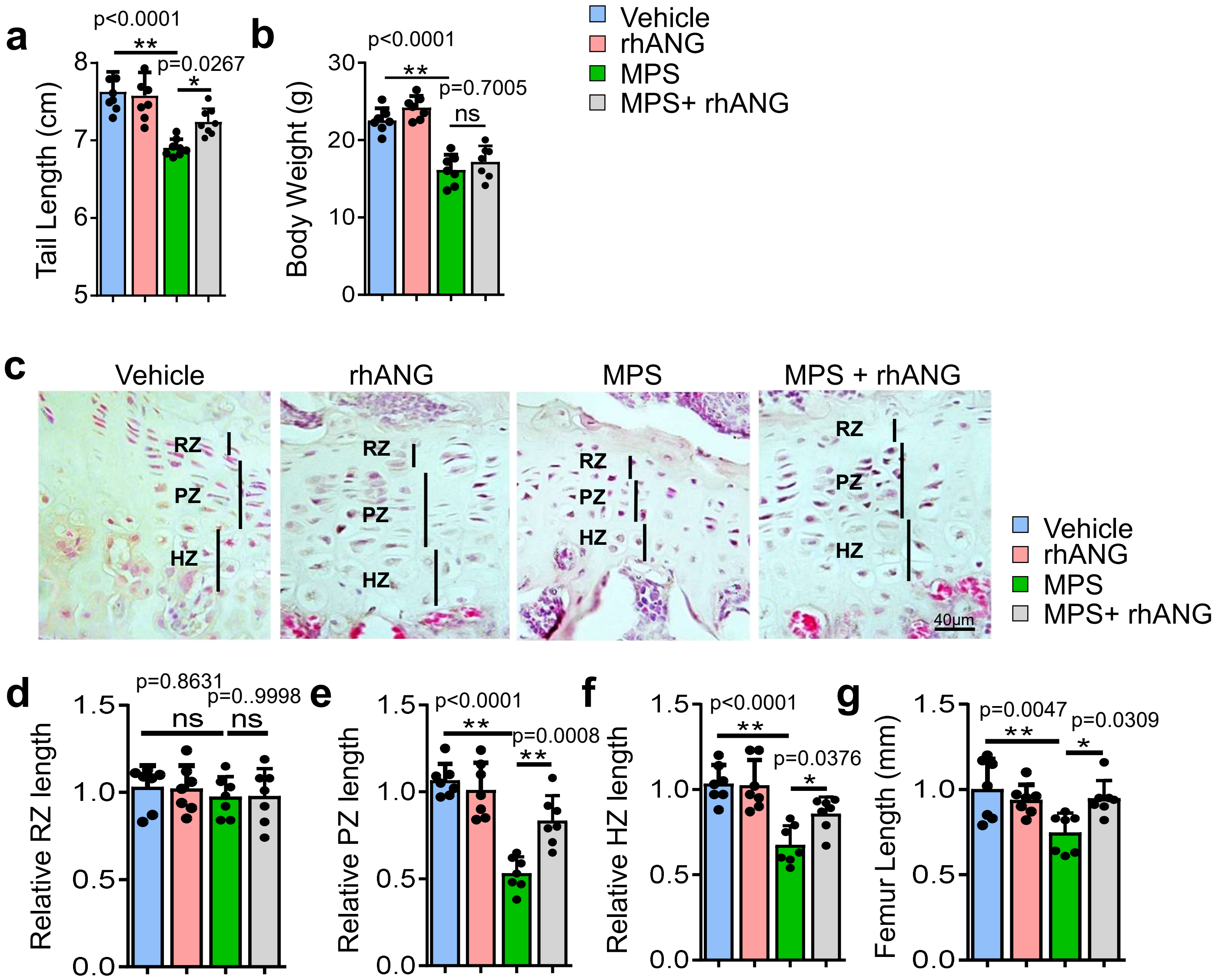

a
b
p<0.0001
p=0.0267
p=0.7005
ns
Tail Length (cm)
Body Weight (g)
Vehicle
rhANG
MPS
MPS+ rhANG
c
Vehicle
rhANG
MPS
MPS + rhANG
RZ
PZ
HZ
40μm
d
e
f
g
p=0.8631
p=0..9998
p=0.0008
p=0.0376
p=0.0047
p=0.0309
Relative RZ length
Relative PZ length
Relative HZ length
Femur Length (mm)

Supplementary Figure 10 rhANG Rescues GC-Induced Growth Retardation Phenotype in Young Mice. (**a-g**) Three-week-old *BALB/c* mice were treated with vehicle, MPS alone at 10 $mg/m^2/day$ or MPS plus rhANG (1 μg/day) by daily intraperitoneal injection for 4 weeks. (**a**) Tail length (cm) was measured each week during the treatment. (**b**) Measurements of body weight in each group. (**c**) Hematoxylin and eosin staining of representative sections of distal femoral growth plates. Measurements of the length of three different chondrocyte zones in the rhANG, MPS and MPS+rhANG group relative to the vehicle control group in (**d**)**,** (**e**) and (**f**), respectively. (**g**) Femur length was calculated by measuring the distance between the apex of femoral head and the most distal point of subchondral bone in distal femur. GP, growth plate; RZ, resting zone; PZ, proliferating zone; and HZ, hypertrophic zone. n=6-10 mice. Data are represented as mean ±s.e.m. *$p < 0.05$, **$p < 0.01$ as determined by one-way ANOVA with post hoc Tukey test.

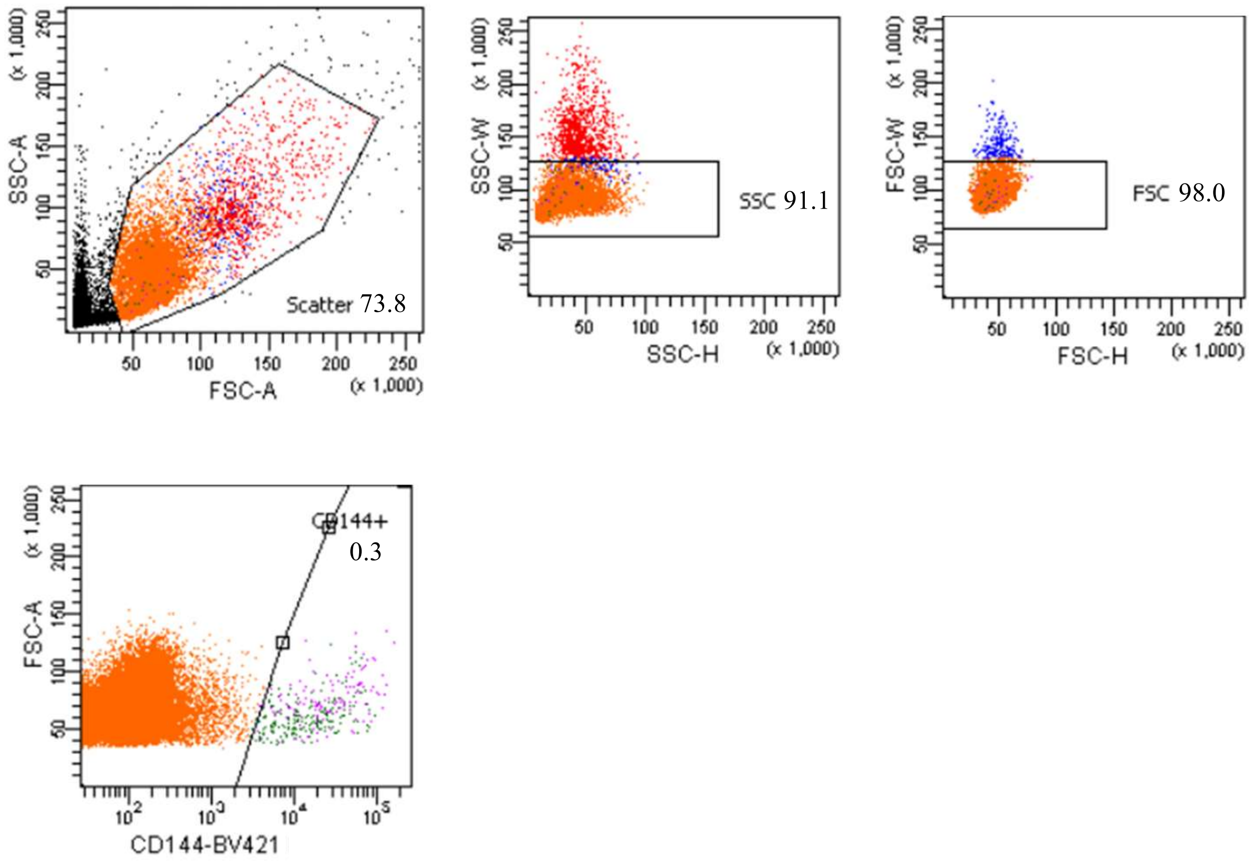

Supplementary Figure 11 Gating strategy for in vivo FACS analysis experiments.

qRT-PCR Primers		
**Gene**	**Forward**	**Reverse**
ki67	ACCGTGGAGTAGTTTATCTGGG	TGTTTCCAGTCCGCTTACTTCT
p16	GAAAGAGTTCGGGGCGTTG	GAGAGCCATCTGGAGCAGCAT
p53	ATCGCCTTCGACATCATCGC	CCCCATGCGTACTCCATGAG
p21	AGAAGGTACTTACGGTGTGGT	GAGAGATTTCCCGAATTGCAGT
ang	TTGATCTTCGTGCTGGGTCTGG	CTCTGTAAGGGCTTCCATTCGC

Supplementary Table 1 Primers used for qRT-PCR.
