# Supplementary material for: Osteoclasts protect bone blood vessels against senescence through the angiogenin/plexin-B2 axis
Source: Nat Commun. 2021 Mar 23;12:1832. doi: 10.1038/s41467-021-22131-1 (PMC7987975; doi:10.1038/s41467-021-22131-1)
Supplement: Supplementary file 3 — Reporting Summary [file 41467_2021_22131_MOESM3_ESM.pdf]

nature research

Corresponding author(s): Mei Wan

Last updated by author(s): Jan 6, 2021

Reporting Summary

Nature Research wishes to improve the reproducibility of the work that we publish. This form provides structure for consistency and transparency in reporting. For further information on Nature Research policies, see our Editorial Policies and the Editorial Policy Checklist.

Statistics

For all statistical analyses, confirm that the following items are present in the figure legend, table legend, main text, or Methods section.

n/a	Confirmed	
☐	☒	The exact sample size (*n*) for each experimental group/condition, given as a discrete number and unit of measurement
☐	☒	A statement on whether measurements were taken from distinct samples or whether the same sample was measured repeatedly
☐	☒	The statistical test(s) used AND whether they are one- or two-sided<br>*Only common tests should be described solely by name; describe more complex techniques in the Methods section.*
☒	☐	A description of all covariates tested
☒	☐	A description of any assumptions or corrections, such as tests of normality and adjustment for multiple comparisons
☐	☒	A full description of the statistical parameters including central tendency (e.g. means) or other basic estimates (e.g. regression coefficient) AND variation (e.g. standard deviation) or associated estimates of uncertainty (e.g. confidence intervals)
☐	☒	For null hypothesis testing, the test statistic (e.g. *F*, *t*, *r*) with confidence intervals, effect sizes, degrees of freedom and *P* value noted<br>*Give P values as exact values whenever suitable.*
☒	☐	For Bayesian analysis, information on the choice of priors and Markov chain Monte Carlo settings
☒	☐	For hierarchical and complex designs, identification of the appropriate level for tests and full reporting of outcomes
☒	☐	Estimates of effect sizes (e.g. Cohen's *d*, Pearson's *r*), indicating how they were calculated

Our web collection on statistics for biologists contains articles on many of the points above.

Software and code

Policy information about availability of computer code

Data collection	Skyscan v1172(Bruker-microCT), ImageJ software v1.49(NIH), Graphpad Prism v7.0(Graphpad software), FACSDiva v8.0.1(BD Bioscience), ZEN v3.2(Zeiss), cellSens v2.2(Olympus), ImageStreamX mark II(Amnis), ImageLab v6.1(Bio-rad), OsteoMeasure v4.00 (OsteoMetrics)
Data analysis	µCT images were reconstructed and analyzed using NRecon v1.6, CTAn v1.9 and CTVol version 2.0 (Bruker MicroCT) (Bruker-microCT). All data analyses were performed using Graphpad Prism v7.0(Graphpad software). ImageJ software v1.49(NIH) was used for quantitative analysis of histology. FACSDiva v8.0.1(BD Bioscience), FlowJo v10.0(BD Bioscience) and IDEAS v6.1 (Amnis) were used to perform Flow cytometry analysis.

For manuscripts utilizing custom algorithms or software that are central to the research but not yet described in published literature, software must be made available to editors and reviewers. We strongly encourage code deposition in a community repository (e.g. GitHub). See the Nature Research guidelines for submitting code & software for further information.

Data

Policy information about availability of data

All manuscripts must include a data availability statement. This statement should provide the following information, where applicable:

- Accession codes, unique identifiers, or web links for publicly available datasets
- A list of figures that have associated raw data
- A description of any restrictions on data availability

All the data supporting the findings of this study are available within the article and its supplementary information files and from the corresponding author upon reasonable request. A reporting summary for this article is available as a Supplementary Information file. The source data underlying western blot in Figures 6 is provided as a Source Data file.

Field-specific reporting

Please select the one below that is the best fit for your research. If you are not sure, read the appropriate sections before making your selection.

☒ Life sciences ☐ Behavioural & social sciences ☐ Ecological, evolutionary & environmental sciences

For a reference copy of the document with all sections, see nature.com/documents/nr-reporting-summary-flat.pdf

Life sciences study design

All studies must disclose on these points even when the disclosure is negative.

Sample size	All animal experiments were performed with enough biological replicates (a minimum of 4) to allow relevant statistical analysis. Sample size was calculated by a power calculation based on microCT effect volume. For baseline animal parameters and in vitro studies sample size was chosen based on our previous experience, experimental approach, availability and feasibility required to obtain statistically significant results.
Data exclusions	All inclusion/ exclusion criteria were pre-established and no samples or animals were excluded from the analysis
Replication	Replication attempts were successfully performed. Immunofluorescence, immunoblots and in vitro studies were performed in three biological replicates, unless indicated differently in the figure legend.
Randomization	The experiments were randomized
Blinding	The experiments were randomized, and the investigators were blinded to allocation during experiments and outcome assessment.

Behavioural & social sciences study design

All studies must disclose on these points even when the disclosure is negative.

Study description	*Briefly describe the study type including whether data are quantitative, qualitative, or mixed-methods (e.g. qualitative cross-sectional, quantitative experimental, mixed-methods case study).*
Research sample	*State the research sample (e.g. Harvard university undergraduates, villagers in rural India) and provide relevant demographic information (e.g. age, sex) and indicate whether the sample is representative. Provide a rationale for the study sample chosen. For studies involving existing datasets, please describe the dataset and source.*
Sampling strategy	*Describe the sampling procedure (e.g. random, snowball, stratified, convenience). Describe the statistical methods that were used to predetermine sample size OR if no sample-size calculation was performed, describe how sample sizes were chosen and provide a rationale for why these sample sizes are sufficient. For qualitative data, please indicate whether data saturation was considered, and what criteria were used to decide that no further sampling was needed.*
Data collection	*Provide details about the data collection procedure, including the instruments or devices used to record the data (e.g. pen and paper, computer, eye tracker, video or audio equipment) whether anyone was present besides the participant(s) and the researcher, and whether the researcher was blind to experimental condition and/or the study hypothesis during data collection.*
Timing	*Indicate the start and stop dates of data collection. If there is a gap between collection periods, state the dates for each sample cohort.*
Data exclusions	*If no data were excluded from the analyses, state so OR if data were excluded, provide the exact number of exclusions and the rationale behind them, indicating whether exclusion criteria were pre-established.*
Non-participation	*State how many participants dropped out/declined participation and the reason(s) given OR provide response rate OR state that no participants dropped out/declined participation.*
Randomization	*If participants were not allocated into experimental groups, state so OR describe how participants were allocated to groups, and if allocation was not random, describe how covariates were controlled.*

Ecological, evolutionary & environmental sciences study design

All studies must disclose on these points even when the disclosure is negative.

Study description	*Briefly describe the study. For quantitative data include treatment factors and interactions, design structure (e.g. factorial, nested, hierarchical), nature and number of experimental units and replicates.*
Research sample	*Describe the research sample (e.g. a group of tagged Passer domesticus, all Stenocereus thurberi within Organ Pipe Cactus National Monument), and provide a rationale for the sample choice. When relevant, describe the organism taxa, source, sex, age range and*

	any manipulations. State what population the sample is meant to represent when applicable. For studies involving existing datasets, describe the data and its source.
Sampling strategy	*Note the sampling procedure. Describe the statistical methods that were used to predetermine sample size OR if no sample-size calculation was performed, describe how sample sizes were chosen and provide a rationale for why these sample sizes are sufficient.*
Data collection	*Describe the data collection procedure, including who recorded the data and how.*
Timing and spatial scale	*Indicate the start and stop dates of data collection, noting the frequency and periodicity of sampling and providing a rationale for these choices. If there is a gap between collection periods, state the dates for each sample cohort. Specify the spatial scale from which the data are taken*
Data exclusions	*If no data were excluded from the analyses, state so OR if data were excluded, describe the exclusions and the rationale behind them, indicating whether exclusion criteria were pre-established.*
Reproducibility	*Describe the measures taken to verify the reproducibility of experimental findings. For each experiment, note whether any attempts to repeat the experiment failed OR state that all attempts to repeat the experiment were successful.*
Randomization	*Describe how samples/organisms/participants were allocated into groups. If allocation was not random, describe how covariates were controlled. If this is not relevant to your study, explain why.*
Blinding	*Describe the extent of blinding used during data acquisition and analysis. If blinding was not possible, describe why OR explain why blinding was not relevant to your study.*

Did the study involve field work? ☐ Yes ☐ No

Field work, collection and transport

Field conditions	*Describe the study conditions for field work, providing relevant parameters (e.g. temperature, rainfall).*
Location	*State the location of the sampling or experiment, providing relevant parameters (e.g. latitude and longitude, elevation, water depth).*
Access & import/export	*Describe the efforts you have made to access habitats and to collect and import/export your samples in a responsible manner and in compliance with local, national and international laws, noting any permits that were obtained (give the name of the issuing authority, the date of issue, and any identifying information).*
Disturbance	*Describe any disturbance caused by the study and how it was minimized.*

Reporting for specific materials, systems and methods

We require information from authors about some types of materials, experimental systems and methods used in many studies. Here, indicate whether each material, system or method listed is relevant to your study. If you are not sure if a list item applies to your research, read the appropriate section before selecting a response.

Materials & experimental systems

n/a	Involved in the study	
☐	☒	Antibodies
☐	☒	Eukaryotic cell lines
☒	☐	Palaeontology and archaeology
☐	☒	Animals and other organisms
☒	☐	Human research participants
☒	☐	Clinical data
☒	☐	Dual use research of concern

Methods

n/a	Involved in the study	
☒	☐	ChIP-seq
☐	☒	Flow cytometry
☒	☐	MRI-based neuroimaging

Antibodies

Antibodies used	We listed the antibodies in the methods section. We included the catalog number for each commercial antibody used, and more detailed Information could be obtained from the companies. We used the following antibodies: Emcn antibody (Santa Cruz, sc-65495, 1:50), APC-conjugated CD31 antibody (R&D SystemsEmcn, FAB3628A, 1:100), BV421-conjugated second antibody against Emcn (BioLegend, 405414, 1: 50),CD144-BV421 (BioLegend, 138013, 1:100) PE-conjugated HMGB1 antibody (BioLegend, 651404, 1:100), Alex594-conjugated Ki67 antibody (Abcam, ab216709), Angiogenin 26-2F antibody(eBioscience, 14-9762-82), mAb17 (kindly provided by Dr. Guo-Fu Hu), PLXNB2 antibody(Proteintech, 10602-1-AP,1:800), AKT antibody (Cell Signaling, 40D4, 1:2000), p-AKT antibody (Cell Signaling, 193H12, 1:1000), ERK1/2 antibody (Cell Signaling, 137F5, 1:1000), p-ERK antibody (Santa Cruz, sc-7383, 1:500), Lamin B1 antibody (Santa Cruz, sc-374015, 1:500), Ki67 antibody (Abcam, ab92742, 1:2000), p21 antibody (Abcam, ab109520,1:2000), GAPDH antibody (Cell Signaling, 14C10, 1:1000), β-Tubulin antibody(Cell Signaling, 9F3, 1:1000), CD31 antibody (R&D Systems, FAB3628G, 1:100), Angiogenin antibody (rabbit monoclonal, C527, 1:200, generated by the laboratory of Dr. Guo-Fu Hu), Plexin-B2 antibody (1:200, eBioscience, eBio3E7), Osterix antibody(Abcam, ab22552, 1:200), Osteocalcin antibody(Takara, M188, 1:200), F4/80 antibody(Abcam, ab6640, 1:200), RFP antibody(Rockland

	antibodies&assays, 600-401-379, 1:200)and HMGB1 antibody (Abcam, ab18256, 1:300) FITC, or Cy3-conjugated secondary antibodies (Jackson ImmunoResearch, 1:200)
Validation	Antibodies were validated in cells as well as in mice bone tissue. We also took into account relevant citations and instructions on manufacturer's websites.

Eukaryotic cell lines

Policy information about cell lines

Cell line source(s)	HUVEC(PCS-100-010, ATCC)
Authentication	Cells were authenticated by examination of classic "cobblestone" morphology and gene expression. Also, all cell lines were carefully labeled and stored until use.
Mycoplasma contamination	Cell line is tested negative for Mycoplasma contamination.
Commonly misidentified lines (See ICLAC register)	No commonly misidentified cell lines were used in the study.

Palaeontology and Archaeology

Specimen provenance	*Provide provenance information for specimens and describe permits that were obtained for the work (including the name of the issuing authority, the date of issue, and any identifying information).*
Specimen deposition	*Indicate where the specimens have been deposited to permit free access by other researchers.*
Dating methods	*If new dates are provided, describe how they were obtained (e.g. collection, storage, sample pretreatment and measurement), where they were obtained (i.e. lab name), the calibration program and the protocol for quality assurance OR state that no new dates are provided.*

☐ Tick this box to confirm that the raw and calibrated dates are available in the paper or in Supplementary Information.

Ethics oversight	*Identify the organization(s) that approved or provided guidance on the study protocol, OR state that no ethical approval or guidance was required and explain why not.*

Note that full information on the approval of the study protocol must also be provided in the manuscript.

Animals and other organisms

Policy information about studies involving animals; ARRIVE guidelines recommended for reporting animal research

Laboratory animals	We used Cdh5-CreERT2 mice (C57 background, stock no. 13073, Taconic Bioscience), BALB/C mice(stock no. 000664, male, 3-weeks old, Jackson Laboratory), C57BL/6J mice (stock no. 000651, male, 3-weeks old, Jackson Laboratory), p16 flox/flox mice(C57 background, generated by Dr. Gloria H. Su's laboratory from the Department of Pathology, Columbia University Medical Center) , p16-tdTom reporter mice (C57 background, male, 3-weeks old, generated by Dr. Norman E. Sharpless's laboratory from the Curriculum in Genetics and Molecular Biology, University of North Carolina School of Medicine). Cdh5-CreERT2 mice were crossed with p16 flox/flox mice to generate Cdh5-CreERT2; p16 flox/flox (p16 iKO, male, 3-weeks old) and p16 flox/flox (WT, male, 3-weeks old) mice. Detailed mouse generation and maintainance were described in detail in the manuscript. Mice were housed in a normal condition with 12:12h light: dark cycle in a temperature-controlled room with food and water ad libitum.
Wild animals	No wild animals were involved in the study.
Field-collected samples	No field collected samples were used in the study.
Ethics oversight	All mice were maintained at the animal facility of The Johns Hopkins University School of Medicine. All experimental protocols (M018M139) were approved by the Animal Care and Use Committee of The Johns Hopkins University, Baltimore, MD.

Note that full information on the approval of the study protocol must also be provided in the manuscript.

Human research participants

Policy information about studies involving human research participants

Population characteristics	*Describe the covariate-relevant population characteristics of the human research participants (e.g. age, gender, genotypic information, past and current diagnosis and treatment categories). If you filled out the behavioural & social sciences study design questions and have nothing to add here, write "See above."*
Recruitment	*Describe how participants were recruited. Outline any potential self-selection bias or other biases that may be present and how these are likely to impact results.*
Ethics oversight	*Identify the organization(s) that approved the study protocol.*

Note that full information on the approval of the study protocol must also be provided in the manuscript.

Clinical data

Policy information about clinical studies

All manuscripts should comply with the ICMJE guidelines for publication of clinical research and a completed CONSORT checklist must be included with all submissions.

Clinical trial registration	*Provide the trial registration number from ClinicalTrials.gov or an equivalent agency.*
Study protocol	*Note where the full trial protocol can be accessed OR if not available, explain why.*
Data collection	*Describe the settings and locales of data collection, noting the time periods of recruitment and data collection.*
Outcomes	*Describe how you pre-defined primary and secondary outcome measures and how you assessed these measures.*

Dual use research of concern

Policy information about dual use research of concern

Hazards

Could the accidental, deliberate or reckless misuse of agents or technologies generated in the work, or the application of information presented in the manuscript, pose a threat to:

No	Yes	
☐	☐	Public health
☐	☐	National security
☐	☐	Crops and/or livestock
☐	☐	Ecosystems
☐	☐	Any other significant area

Experiments of concern

Does the work involve any of these experiments of concern:

No	Yes	
☐	☐	Demonstrate how to render a vaccine ineffective
☐	☐	Confer resistance to therapeutically useful antibiotics or antiviral agents
☐	☐	Enhance the virulence of a pathogen or render a nonpathogen virulent
☐	☐	Increase transmissibility of a pathogen
☐	☐	Alter the host range of a pathogen
☐	☐	Enable evasion of diagnostic/detection modalities
☐	☐	Enable the weaponization of a biological agent or toxin
☐	☐	Any other potentially harmful combination of experiments and agents

ChIP-seq

Data deposition

☐ Confirm that both raw and final processed data have been deposited in a public database such as GEO.

☐ Confirm that you have deposited or provided access to graph files (e.g. BED files) for the called peaks.

Data access links<br>*May remain private before publication.*	*For "Initial submission" or "Revised version" documents, provide reviewer access links. For your "Final submission" document, provide a link to the deposited data.*
Files in database submission	*Provide a list of all files available in the database submission.*
Genome browser session<br>(e.g. UCSC)	*Provide a link to an anonymized genome browser session for "Initial submission" and "Revised version" documents only, to enable peer review. Write "no longer applicable" for "Final submission" documents.*

Methodology

Replicates	*Describe the experimental replicates, specifying number, type and replicate agreement.*

Sequencing depth	*Describe the sequencing depth for each experiment, providing the total number of reads, uniquely mapped reads, length of reads and whether they were paired- or single-end.*
Antibodies	*Describe the antibodies used for the ChIP-seq experiments; as applicable, provide supplier name, catalog number, clone name, and lot number.*
Peak calling parameters	*Specify the command line program and parameters used for read mapping and peak calling, including the ChIP, control and index files used.*
Data quality	*Describe the methods used to ensure data quality in full detail, including how many peaks are at FDR 5% and above 5-fold enrichment.*
Software	*Describe the software used to collect and analyze the ChIP-seq data. For custom code that has been deposited into a community repository, provide accession details.*

Flow Cytometry

Plots

Confirm that:

☒ The axis labels state the marker and fluorochrome used (e.g. CD4-FITC).

☒ The axis scales are clearly visible. Include numbers along axes only for bottom left plot of group (a 'group' is an analysis of identical markers).

☒ All plots are contour plots with outliers or pseudocolor plots.

☒ A numerical value for number of cells or percentage (with statistics) is provided.

Methodology

Sample preparation	the epiphysis was removed from the distal femora and proximal tibia, and only the metaphyseal region was processed. The bones were then crushed in ice-cold PBS with a mortar and pestle. Whole bone marrow was digested with collagenase A (Sigma, 11088793001, 2 mg/mL) and trypsin (2.5 mg/mL) in PBS at 37°C for 20 minutes to obtain single-cell suspensions.
Instrument	FACSCalibur instrument
Software	FACSDiva (BD Biosciences)
Cell population abundance	Between 10,000 and 100,000 cells were acquired per sample, and the total population was analysed.
Gating strategy	From the starting cell population, the cells were gated by: FSC-A/SSC-A; FSC-A/FSC-H followed by gating CD144-BV421-A/tdTOM-A for analysis of senescent vascular cells in p16-tdTOM mice; tdTOM-A/FSC-A for analysis of senescent cells in p16-tdTOM mice; CD144-BV421-A/SA-βGal-FITC-A for analysis of senescent vascular cells in wild type mice and Emcn-BV421-A/CD31-APC-A for analysis of vessel change in wild type mice.

☒ Tick this box to confirm that a figure exemplifying the gating strategy is provided in the Supplementary Information.

Magnetic resonance imaging

Experimental design

Design type	*Indicate task or resting state; event-related or block design.*
Design specifications	*Specify the number of blocks, trials or experimental units per session and/or subject, and specify the length of each trial or block (if trials are blocked) and interval between trials.*
Behavioral performance measures	*State number and/or type of variables recorded (e.g. correct button press, response time) and what statistics were used to establish that the subjects were performing the task as expected (e.g. mean, range, and/or standard deviation across subjects).*

Acquisition

Imaging type(s)	*Specify: functional, structural, diffusion, perfusion.*
Field strength	*Specify in Tesla*
Sequence & imaging parameters	*Specify the pulse sequence type (gradient echo, spin echo, etc.), imaging type (EPI, spiral, etc.), field of view, matrix size, slice thickness, orientation and TE/TR/flip angle.*
Area of acquisition	*State whether a whole brain scan was used OR define the area of acquisition, describing how the region was determined.*

Diffusion MRI ☐ Used ☐ Not used

Preprocessing

Preprocessing software	*Provide detail on software version and revision number and on specific parameters (model/functions, brain extraction, segmentation, smoothing kernel size, etc.).*
Normalization	*If data were normalized/standardized, describe the approach(es): specify linear or non-linear and define image types used for transformation OR indicate that data were not normalized and explain rationale for lack of normalization.*
Normalization template	*Describe the template used for normalization/transformation, specifying subject space or group standardized space (e.g. original Talairach, MNI305, ICBM152) OR indicate that the data were not normalized.*
Noise and artifact removal	*Describe your procedure(s) for artifact and structured noise removal, specifying motion parameters, tissue signals and physiological signals (heart rate, respiration).*
Volume censoring	*Define your software and/or method and criteria for volume censoring, and state the extent of such censoring.*

Statistical modeling & inference

Model type and settings	*Specify type (mass univariate, multivariate, RSA, predictive, etc.) and describe essential details of the model at the first and second levels (e.g. fixed, random or mixed effects; drift or auto-correlation).*
Effect(s) tested	*Define precise effect in terms of the task or stimulus conditions instead of psychological concepts and indicate whether ANOVA or factorial designs were used.*

Specify type of analysis: ☐ Whole brain ☐ ROI-based ☐ Both

Statistic type for inference (See Eklund et al. 2016)	*Specify voxel-wise or cluster-wise and report all relevant parameters for cluster-wise methods.*
Correction	*Describe the type of correction and how it is obtained for multiple comparisons (e.g. FWE, FDR, permutation or Monte Carlo).*

Models & analysis

n/a	Involved in the study
☐	☐ Functional and/or effective connectivity
☐	☐ Graph analysis
☐	☐ Multivariate modeling or predictive analysis

Functional and/or effective connectivity	*Report the measures of dependence used and the model details (e.g. Pearson correlation, partial correlation, mutual information).*
Graph analysis	*Report the dependent variable and connectivity measure, specifying weighted graph or binarized graph, subject- or group-level, and the global and/or node summaries used (e.g. clustering coefficient, efficiency, etc.).*
Multivariate modeling and predictive analysis	*Specify independent variables, features extraction and dimension reduction, model, training and evaluation metrics.*
